# Exploring a Shared History of Colonization, Historical Trauma, and Links to Alcohol Use With Native Hawaiians: Qualitative Study

**DOI:** 10.2196/68106

**Published:** 2025-07-29

**Authors:** Cynthia Taylor Greywolf, Donna Marie Palakiko, Pallav Pokhrel, Elizabeth A Vandewater, Merle Kataoka-Yahiro, John Casken

**Affiliations:** 1 School of Nursing and Dental Hygiene, University of Hawai'i at Mānoa, 2528 McCarthy Mall, Room 439, Honolulu, HI, 96822, United States, 1 8089564865; 2 University of Hawai'i at Mānoa Cancer Center, Honolulu, HI, United States; 3 Cain Center for Nursing Research, School of Nursing, University of Texas at Austin, Austin, TX, United States

**Keywords:** historical trauma, intergenerational trauma, Indigenous health, alcohol use, substance misuse, health disparity

## Abstract

**Background:**

Most studies using historical trauma theory have focused on American Indian tribes. There remains a dearth of research exploring historical trauma and substance use among Native Hawaiians. Native Hawaiians and American Indians experience a startlingly high degree of physical and mental health disparities and alcohol and other substance misuse. Indigenous scholars posit that historical trauma is intergenerationally transmitted to subsequent generations and is the primary cause of today’s health and substance use disparities among these Indigenous populations.

**Objective:**

This study aimed to explore the lived experiences of colonization, historical trauma, and alcohol use among Native Hawaiians living in rural Hawaii.

**Methods:**

This qualitative study was guided by Husserl’s transcendental phenomenological design. The historical trauma conceptual framework and story theory guided the study. The Native Hawaiian Talk-Story method was used to collect data from 10 Native Hawaiian adult participants in one-to-one interviews. The modified Stevick-Keen-Colaizzi method was used for data analysis.

**Results:**

In total, four themes emerged: (1) alcohol did not exist in Hawaii before European explorers arrived; (2) alcohol helped expand colonialism in Hawaii; (3) alcohol is used today as a coping strategy for feelings of grief and anger over losses (land, people, cultural traditions, and language); and (4) the kupuna (elders) teach the younger generations to drink alcohol.

**Conclusions:**

Native Hawaiians, like American Indians, experienced historical trauma, which is transmitted intergenerationally, resulting in mental and physical health disparities, substance misuse, and feelings of discrimination. The introduction of alcohol by European explorers provides the foundation for problematic alcohol use among Native Hawaiians today.

## Introduction

### Background

Alcohol was the first psychoactive drug introduced to Native Hawaiians in 1778 by European explorers. The introduction of other varieties of alcohol quickly followed as the foreigners arrived and settled in Hawaii [1]. Historical accounts reveal that alcohol was weaponized and used as a tool against Indigenous populations during the colonization of Indigenous lands. The introduction of alcohol resulted in cultural shifts to alcohol use where it once did not exist and where it has now become normalized [2]. Due to its addictive and intoxicating properties, alcohol had devastating consequences among Indigenous peoples [1,3-6]. Historically traumatic events leading to the loss of people, land, traditions, and language that occurred during the colonization of Indigenous lands left in its wake mass group trauma experiences and psychological wounds among Indigenous people, the consequences of which continue today. The introduction of alcohol to Indigenous people by early European explorers and colonizing settlers provided the foundation for problematic alcohol use among them today [1,3,5].

Today’s Native Hawaiians, American Indians, and Alaska Natives are the contemporary descendants of the original Indigenous peoples who occupied lands before the arrival of European explorers and settlers and the establishment of the United States. American Indians and Alaska Natives are defined explicitly as citizens of sovereign federally recognized tribes and groups with nation-to-nation relationships with the US government. Notably, Indigenous peoples, before colonization, were healthy [7,8]. However, Native Hawaiians, American Indians, and Alaska Natives in the United States today experience disproportionately higher rates of physical and mental health disparities and significantly higher rates of cigarette smoking, alcohol, and other substance use, suicide rates, and traumatic exposure than other ethnic groups in the state [2,3,6,9-15].

In Hawaii, Native Hawaiians are at significantly greater risk for adverse health outcomes and high-risk health behaviors as compared with other ethnic groups [6,9]. Recent reports highlight high rates of substance use; 47% of Native Hawaiian adults report current alcohol and tobacco use, and 35% report lifetime substance use, including cannabis and opioids. Rates of alcohol use disorder, depression, and generalized anxiety disorder prevalence have been reported as 27%, 27%, and 19%, respectively. Finally, approximately 30% of Native Hawaiian adults report past-year treatment needs for lifetime illicit substance abuse [14]. Despite the high risk for alcohol and substance use, little is known about the risk factors leading to alcohol use and misuse that are unique to Native Hawaiians today [1,3].

Indigenous scholars have hypothesized that historical trauma is a primary cause of social stress, mental health disparities, and problems with alcohol and substance use among Indigenous peoples today [3,6,9,11,16-23]. Much research has focused on the links between historical trauma and its impacts. However, these studies have focused mainly on American Indians. There is a need to fill the gap in research by exploring whether these links are present in the Native Hawaiian populations. There is scant research specifically on Native Hawaiians’ experiences related to historical trauma [3,6]. Although there is cultural diversity among Indigenous tribes and groups, significant similarities exist in the events that led to historical trauma and the ways that the historical trauma response manifests [2,3,6,24]. Problematic alcohol use among American Indians has been defined as a historical trauma response to the emotional and psychological stressors resulting from colonization [2]. To address this gap in the literature, historical trauma theory was used as a conceptual model for this study [17,24].

### Historical Trauma

Historical trauma is defined as the emotional and psychological wounding over the lifespan and across generations from mass group trauma experiences that are associated with the historical losses of land, people, language, and culture of colonization by an outside group. What is unique about historical trauma is its widespread impact on Indigenous populations, which was done with a purposeful and destructive intent that resulted in the collective suffering of the primary generation and then transmitted to present-day descendants, making this a form of trauma exceptionally devastating for individuals, families, and communities [2,16,23,24]. The historical trauma response is a constellation of physical, psychological, and sociological symptoms experienced by the descendants [16,17]. At the individual level, it includes adverse childhood events, mental health disparities, and problematic alcohol use; at the familial level, parental stress and problematic alcohol use; and at the community level, the breakdown of cultural customs and traditions and problematic alcohol use in the community [2,16,17]. The consequences of the historical trauma response can be transmitted intergenerationally as descendants continue to identify with ancestral pain [16,17]. Historically unresolved grief is an element of this response. It is the profound, unsettling grief resulting from cumulative losses that are compounded by the prohibition of Indigenous cultural traditions and practices [23]. The aim of this qualitative study was to explore the impact of historical trauma on the lived experiences of Native Hawaiians and the use of alcohol in a rural setting.

### Historical Trauma Conceptual Model

**Figure 1.**
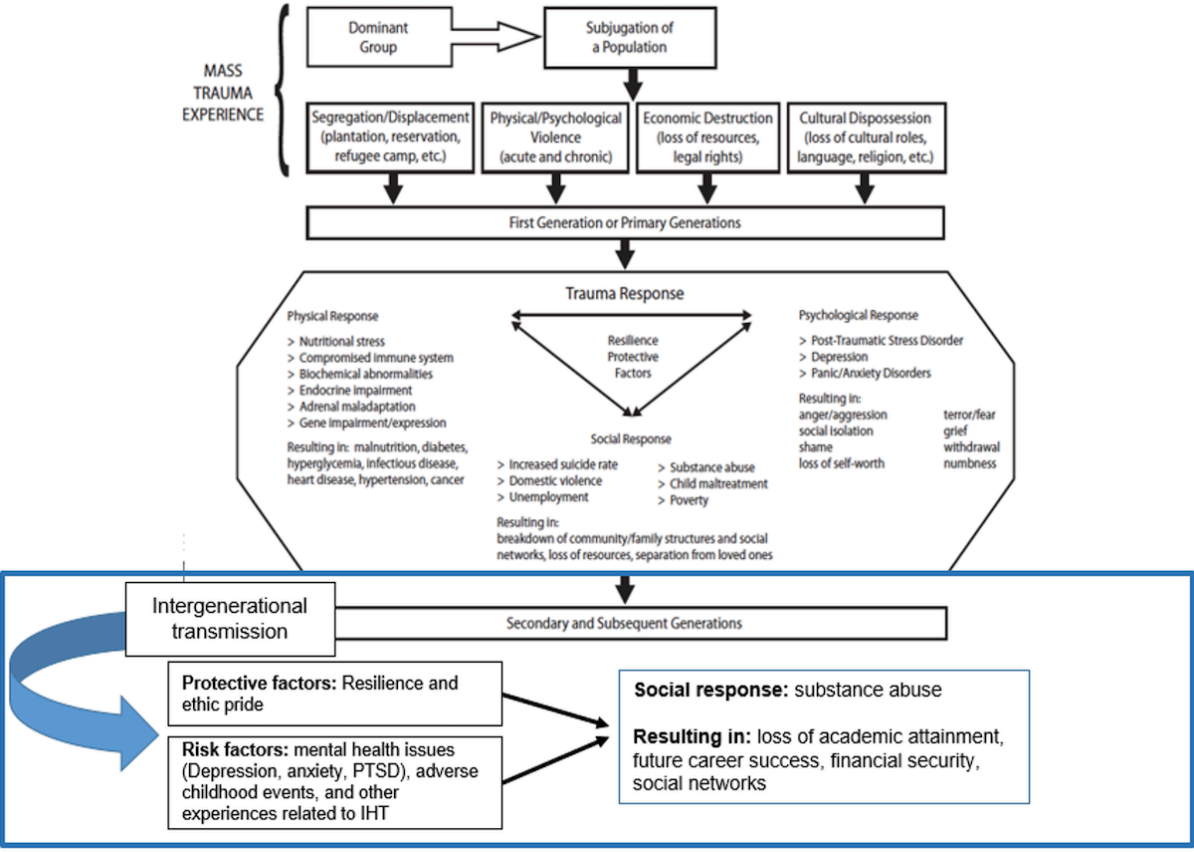
Conceptual model adapted from Sotero’s historical trauma conceptual model [17]. PTSD: post-traumatic stress disorder.

The historical trauma conceptual framework provides an explanatory model of how historical trauma originated and was transmitted through intergenerational transmission to succeeding generations and links to mental health disparities (risks and protective factors) and problematic alcohol use, which is the focus of this study (Figure 1) [17].

## Methods

### Study Design

Considering Indigenous ontology and epistemology, a qualitative transcendental phenomenological approach was identified as the best for this study as it empowered the Native Hawaiian participants to share their day-to-day experiences of historical trauma’s impact on their daily lives [25]. This approach is grounded in Edmund Husserl’s philosophy and was founded on comprehensive descriptions of meanings, perceptions, and experiences of the phenomena under study and was selected by the principal investigator (CTG) who was able to describe her own experiences with the phenomenon under study through the same process as all of the participants. This process revealed any underlying biases and established the ability to look at the data with a fresh, unbiased eye.

### Sample and Procedure

A local Native Hawaiian community member assisted with the recruitment of the 10 Native Hawaiian adult participants, which involved purposive nonprobability sampling in a single rural community. The study was not advertised but was disseminated by word of mouth. The participants did not want to publicly disclose their location in Hawaii for fear of identification and stigmatization. The inclusion criteria were (1) Native Hawaiians living in Hawaii who self-identify as full or part Native Hawaiian, (2) aged 30 to 60 years, (3) able to speak fluent English, (4) willing to have a face-to-face interview that was audio-recorded and, (5) willing to provide perspectives related to the topics under study. The generations between 30 and 60 years of age were selected as they could reflect on both the children and grandparent generations’ perceptions of colonization, historical trauma, and problematic alcohol use. Individuals younger than 30 years and older than 60 years were excluded. Data saturation was reached with 10 participants, which was determined to be an adequate sample size for the following reasons: (1) the analysis was able to reveal clear interpretations of the data, (2) there were no new findings with further interviews, and (3) the narratives became redundant [26,27]. The sample size for this study was large enough to acquire enough data to describe the phenomenon of interest sufficiently and to address the research questions. This was in keeping with the suggestion by Glaser and Strauss [27] that saturation is a criterion for determining the appropriate sample size in a qualitative study.

The study’s trustworthiness was established using credibility, transferability, dependability, and confirmability [28]. The principal investigator (CTG), a Cherokee Nation citizen who lived in the community, was invited to meet with the kupuna (elders) weekly at a local community health center to talk about the cultural and health-related similarities and differences between the Cherokee and Native Hawaiians. These meetings continued for 2 years before the study was conducted and helped to establish a relationship with the Native Hawaiian community. These relationships ensured the cultural validity of the research findings and prevented any further harm that previous researchers within this community may have caused in the past.

A semistructured script of open-ended questions was used to conduct the interviews (Multimedia Appendix 1). Interviews were conducted using the Talk Story methodology—a culturally specific storytelling method used by Native Hawaiians [22,29]. Individual face-to-face interviews were chosen because it is more likely that the participants would be forthcoming in face-to-face interviews. The interviews took place in August 2019 at mutually agreed-upon private, comfortable, and quiet locations. As the interviews were conducted, no time restraints allowed the participants to tell their stories. The interviews lasted from 60 minutes to 80 minutes. At the beginning of each interview, information was given about why the research was being conducted, along with biases and assumptions. The participants were compensated with a US $25 gift card. The interviews were recorded and transcribed verbatim using NVivo version 10 (Lumivero). The guiding interview questions included, (1) can you share any stories about alcohol use in Hawaii before Captain Cook arrived in Hawaii, (2) can you share any stories about the kupuna (elders) and alcohol use when they were growing up and you were growing up, and (3) can you share any stories of how alcohol is used by Native Hawaiians in your area currently?

### Data Analysis

The first and sixth authors (CTG and JC) analyzed the transcripts independently and agreed greater than 80% of the time. Thematic analysis was used to interpret the transcripts using the Modified Stevick, Colaizzi, Keen method, which dictates that the researcher collecting the data should be one of the participants of the study [25]. The principal investigator (CTG) described her own experiences with the phenomenon under study through the same process as all of the participants described below. This process revealed any underlying biases and established the ability to look at the data with a fresh, unbiased eye. This method followed an inductive approach, forming theoretically driven interpretations of the meanings identified in each transcript. This method allowed for the in-depth exploration of all participants’ subjective experiences of the phenomenon of interest of the study. Each transcript was reviewed many times to determine the unchanging horizons, the invariant constituents, and the meaning units to identify the initial codes and, finally, the significant core themes. Triangulation was achieved by comparing the participants’ transcripts to one another and the literature review. The rigor of the study was enhanced by triangulation and member checking. Then, the analyses established associations and patterns of meaning across the initial notes within each transcript, contributing to the initial codes and themes.

The initial steps of the analysis included epoche or bracketing, which is temporarily suspending assumptions and biases in order to focus on the phenomenon of interest, and reduction of the data including (1) horizonalization or the determination of the unchanging horizons, the invariant constituents, or the meaning units, (2) identification and clustering of core themes, and (3) the construction of core themes using verbatim dialogue from the participants’ stories. The final steps of the analysis included, first, the construction of individual textual descriptions (what occurred) from the stories of the participants. Second, the construction of an individual composite textural description from the stories of all of the participants. Third, the construction of individual and composite structural descriptions (how it occurred) underlying the participants’ experiences, including the following structures: (1) universal structure of time, (2) universal structure of space, (3) universal structure of bodily concerns, (4) universal structure of materiality, (5) universal structure of causality, (6) universal structure of self-in-relation, and (7) universal structure of relation-to-others. Finally, it included the synthesis of meanings and the construction of a universal or a composite textural and structural description of the participants’ experiences. In conclusion, a universal or composite textural-structural description from all of the participants’ stories was completed through imaginative variation by synthesizing and integrating the individual textual-structural descriptions into a universal or composite description of the participants’ experiences represented as a whole [25].

### Ethical Considerations

The University of Hawaiʻi at Mānoa Institutional Review Board approved the protocol for this study (protocol 2019‐00414). Each participant was informed of the purpose of the study before any interviews were conducted. All of the participants provided written informed consent to participate. Efforts were undertaken to maintain the participants’ privacy and confidentiality. The consent was explained in detail, outlining the risks and benefits of the study and that they were participating in the study voluntarily and could withdraw their consent at any time.

## Results

### Overview

The participants primarily identified as female, aged 50-59 years, were married or partnered, and were employed (Table 1).

**Table 1.** Demographic information of participants (N=10).

Characteristic	Value
Race or ethnicity, n (%)	
Native Hawaiian	10 (100)
Age (years), mean (range)	38 (30-59)
Age (years), n (%)	
30-39	3 (30)
40-49	2 (20)
50-59	5 (50)
Identified gender, n (%)	
Male	2 (20)
Female	8 (80)
Marital status, n (%)	
Divorced	2 (20)
Married or partnered	8 (80)
Employment status, n (%)	
Employed for wages	7 (70)
Self-employed	3 (30)

The final qualitative themes that emerged from the analysis included: (1) alcohol did not exist in Hawaii before European explorers arrived, (2) alcohol helped expand colonialism in Hawaii, (3) alcohol is used today as a coping strategy for feelings of grief and anger over losses (land, people, cultural traditions, and language), and (4) the kupuna teach the younger generations to drink alcohol. Rich descriptions and illustrative quotes are provided further in this study and in Multimedia Appendix 2.

### Theme 1: Alcohol Did Not Exist in Hawaii Before Explorers Arrived

The participants described their perceptions related to when Native Hawaiians were first exposed to alcohol. One participant reported:

*The thing I know is, I don’t think we had alcohol before Captain Cook.*

Other participants noted:

*Alcohol was introduced by Europeans. The historical accounts are that it was deliberately introduced once its impact on Indians was understood.*

Several other participants described how okolehao (a mildly alcoholic brew made from the ti plant native to Hawaii) was introduced to Native Hawaiians by European explorers:

*I don’t think we had alcohol...but the introduction of okolehao started Native Hawaiians’ problems with alcohol. Before, you know, we had okolehao, which was alcohol. I know we didn’t have it before Captain Cook, though...*

Others described that before European explorers arrived, “awa” was used (“awa” is a nonalcoholic, nonnarcotic, and mildly psychoactive substance):

*What I know is that we had awa, but I don’t think we had alcohol…*

Many of the participants described that alcohol was deliberately introduced to Native Hawaiians.

*Alcohol is a legacy that continues to affect the quality of life for us.*

Another participant expressed:

*If he [Captain Cook] didn’t come, we wouldn’t have the issues we have today...there would be no alcohol.*

### Theme 2: Alcohol Helped Expand Colonialism in Hawaii

Most of the participants described their beliefs that alcohol expanded colonialism in Hawaii. This caused frustration among the participants, and many noted the sadness related to the introduction of alcohol, how it was used to expand colonialism in Hawaii, which has contributed to the ongoing alcohol use problems they have today. One participant noted that they were:

*...feeling frustrated that the US does not care about managing alcohol use in Native Hawaiians because they have gained control of the land.*

Another noted:

*...the kupunas [elders] say that when our queen was overthrown, and the lands were taken away, it was taken away with the use and trade of rum and alcohol.*

Another noted:

*The kupunas drink because of this [the loss of land], and now a lot of people drink because of what we lost and that we drink the most beer in the state of Hawaiʻi because it is cheaper than anywhere else in Hawaiʻi here.*

Multiple participants recognized that alcohol and the regulation of alcohol by settlers were used to help overthrow the Native Hawaiian monarchy. For example, one described that,

*...alcohol and the regulation of alcohol by the colonizers before Hawaiʻi became a state was used as a means to help overthrow our monarchy.*

Another stated:

*The regulation of alcohol by them [colonizers] before Hawaiʻi became a state was used to help overthrow the monarchy.*

Finally, one participant stated that,

*After 1893, look at what happened….you could see there’s a history…with alcohol at that point with the laws. Right? But what happened after that it was like they didn’t care anymore because they already got the land. Right?*

### Theme 3: Alcohol is Used Today as a Coping Strategy for Feelings of Grief and Anger Over Losses (Land, People, Cultural Traditions, and Language)

Participants shared their experiences about the use of alcohol in their community. They reported that many Native Hawaiian people in their community “are alcoholics.” Several expressed that

*Native Hawaiians drink alcohol to numb feelings of low self-esteem and being pushed down.*

Others shared that diminished self-esteem and grief related to the loss of land, people, cultural traditions, and language. Participants clearly articulated how alcohol was used as a coping mechanism and as self-medication. For example, one participant shared:

*yeah, I think many Hawaiians drink….because of emotional pain because we Hawaiians lost so much, and I think that some just party with it. I really believe so a lot.*

Another participant said:

*I believe that especially with the men too. Right? Hawaiians, they’re pushed down, so they use alcohol. I just recently lost my fiancée, and he had a bad upbringing, and he dealt with a lot of demons. So, he did drugs… you do all of that because once somebody strips your identity, who you are, then it’s hard because it brings down the self-esteem.*

Later, this participant went on to say:

*When you get people always knocking you down, it’s hard, and I see that a lot in our community… and that’s what they [men especially] do to cope is use alcohol or drugs.*

Several also noted that this lingering pain and the use of substances then leads to anger, which can also be expressed as violence. For example, one stated:

*I think some of them [Native Hawaiians] drink because they are angry about what’s happened to us Hawaiians.*

Another stated:

*Alcohol is a really big problem here, which causes a lot of violence because people bottle things up…and so, when people are in pain, they drink more.*

### Theme 4: The Kupuna (Elders) Teach the Younger Generations to Drink Alcohol

When asked to share how Native Hawaiians currently use alcohol in their community and if the kupuna ever talked about how alcohol was used when they were growing up, several of the participants reported that it is the kupuna who are teaching the younger generations how to drink alcohol. One participant stated that

*...the younger generation is like, the older generation in how they drink.*

Another participant reported:

*...the kupunas have taught the younger generations to drink alcohol.*

She expressed concerns “...because…she continues to see them drinking.”

Another participant reported that:

*Alcohol is sold at the cheapest in all islands in this area…Native Hawaiians love alcohol.*

Several participants noted concerns about seeing many of the school-age kids drinking and using methamphetamine, and about the impact of these substances on them, and feel that the kids learn to drink when they see the kupuna drinking. One of the participants expressed feeling that,

*Some of the kupunas do not think that drinking is a problem… but feels that methamphetamine is becoming a bigger problem among Native Hawaiians than alcohol.*

One expressed that,

*Our kupunas teach us how to drink. It’s what our kupunas are doing—teaching the younger generation how to drink alcohol and be like that. Yeah, look when I say habit my mom and dad drank. Drinking was considered no problem whatsoever. However, there was a problem because as we fast forward with what some those kupuna were doing destroyed our family.*

Another participant stated:

*There are a lot of alcoholics, especially in my family. We grew up around it.*

Another participant stated:

*I have to put that on my family, and I would say it’s just a cycle like you know with my grandfather, and my uncle, who I would say is seventy. It’s just normal to just drink, drink, drink.*

Later, she commented that,

*...they [her grandfather and uncle] wake up wasted and drink again the next day. So, I think it’s something when I look at that; it has been passed down.*

## Discussion

### Principal Findings

Participants in this study shared stories which supported the following four themes: (1) alcohol did not exist in Hawaii before European explorers arrived, (2) alcohol helped expand colonialism in Hawaii, (3) alcohol is used today as a coping strategy for feelings of grief and anger over losses (land, people, cultural traditions, and language), and (4) the kupuna (elders) teach the younger generations to drink alcohol. These themes were supported in the literature describing experiences both in Hawaii as well as among American Indian communities.

### Alcohol, the Earliest Psychoactive Drug Introduced in Hawaii

Participant recollections of the history of the introduction of alcohol were in line with the literature. A review of the historical records revealed that the first documented accounts of alcohol (grog, ie, rum and water) appeared in Hawaii in 1778. By 1790, European explorers had introduced okolehao (iron bottom), a mild fermented brew made from the ti plant, native to Hawaii, and distilled in crude cast iron pots. The first Native Hawaiian chief to buy rum was in 1791. By the early 1800s, Native Hawaiian chiefs regularly drank gin, brandy, vodka, rum, and okolehao, and by 1810, excessive alcohol consumption had become a habit of many of the Native Hawaiian chiefs [1,5]. Alcohol was the earliest modern psychoactive drug to be introduced to Native Hawaiians and was imported in large quantities during the 19th century. Native Hawaiians quickly became addicted to alcohol, which had rapidly taken over from the traditional use of awa. The use of awa was prevalent in Hawaii long before European contact; awa was the only substance known in Hawaii. It was considered sacred, and its use was highly regulated by strict ceremonial rules, making abuse difficult [1,3,5]. Traditional Native Hawaiian culture had no perception of psychoactive addiction because awa lacked the neurobiological chemistry to create that effect. Awa is “non-narcotic, non-opiate, non-alcoholic, non-fermented, non-hallucinogenic, and is not physiologically addictive” [1]. Native Hawaiians quickly became addicted to alcohol, which had rapidly taken over from awa as the drug of choice [1].

### A Shared History With American Indians

The history is the same with American Indian tribes, where distilled alcohol did not exist before colonization. Some tribes produced weak fermented beverages that were used mostly for ceremonial practices [3]. Today, contemporary stereotypes, such as the drunken Indian, continue to impact the mental health of Indigenous peoples. All too often, quick assumptions are made associating Indigenous people with alcoholism and poverty. Indigenous peoples continue to struggle to make meaning of their identity and place in the world. Issues, such as racism, discrimination, and marginalization, have worsened mental health, and adverse historical events are associated with substance use and mental health issues and unresolved grief [4,16].

### The Impact of Colonization on Identity and the Regulation of Alcohol Among Native Hawaiians and American Indians

The introduction and regulation of alcohol played a considerable role in the colonization of Hawaii, as it had with American Indian tribes, by destabilizing sovereignty and facilitating the expansion and advancement of the colonial mission. European settlers intentionally disrupted Native Hawaiian ontology and epistemology. Native Hawaiians understood identity through genealogical, kinship ties, and place of birth. As with American Indians, the missionaries introduced Christianity, which taught new ways of thinking, including new patriarchal norms and the concepts of race and class [30-32]. These new concepts replaced traditional Native Hawaiian concepts. European settlers managed to influence Native Hawaiians to advance their own interests [23,32-35]. Liu and Alameda [8] stated, “the violence permeated every level, from disruption of traditional ontology and epistemology and violent displacement with Christianity and other Western systems to the appropriation of lands, loss of traditional economy and ultimately the loss of self-government.” The factors giving rise to this include health impacts on the Native Hawaiians after contact with European settlers and their deliberate attempts to shape Native Hawaiian society to their own interests.

During the late 18th and early 19th centuries, infectious diseases were introduced to Native Hawaiians, and the population was reduced by 84% in 60 years. This allowed Native Hawaiians to be displaced from large tracts of land they had farmed and lived on for generations, converting them from self-sufficiency to dependence on outsiders [1]. Some Native Hawaiians tried maintaining cultural and religious traditions, while others adopted Christianity. The contradictions between the Native Hawaiian disrupted traditional spiritual practices and Christianity connect with how they conceptualized illness and the enticement to use alcohol because of its intoxicating effects as a more effective means of self-medicating in times of enormous losses. For example, when the missionaries arrived in 1820, they raised concerns over whether the Native Hawaiian monarchy could manage alcohol use and public disorder, which interfered with commerce and trade. These concerns influenced the Native Hawaiian monarch to transition to Christian laws, first, as oral declarations concerning everyone to regulate alcohol use [5]. The critique of Native Hawaiians’ use of alcohol was part of the larger colonial discussion, suggesting that they were not capable of political self-governance. Drinking by Native Hawaiians was considered a problem, but not in the same way that drinking was a problem for Whites. The Native Hawaiians’ use of alcohol was increasingly considered a threat to social order, although White foreign sailors and merchants’ use of alcohol demonstrated unwanted behaviors as well. Because of these views, a unitary system of laws with dual legal codes for alcohol use was established, criminalizing alcohol use in Native Hawaiians but not in Whites. The regulation of alcohol with Native Hawaiians contrasted with the circumstances of alcohol regulation with American Indians only in that the Native Hawaiian monarchy was influenced by the American missionaries to adopt the same prohibition policies that the US government established to control drinking among American Indians. The salient point from this is that the regulation of alcohol among both American Indians and Native Hawaiians was a means of acquiring and maintaining political autonomy over them, which further eroded cultural traditions and power, resulting in cultural shifts using alcohol [5,35-37].

### Alcohol as a Way of Coping

In addition to participants acknowledging cultural loss and historical trauma, they shared with American Indians and the use of alcohol as a way to cope. Native Hawaiian mental health was traditionally related to pono, a state of balance attained through proper behaviors with the environment, the people, and the spiritual realm. The cause of illness was believed to be rooted in the spirit instead of the physical body. Alcohol’s intoxicating effects were found to be better at self-medicating than awa, when so many losses were occurring. Alcohol had become widely used to self-medicate and quickly displaced awa. These observations are consistent with research findings from a scoping review on American Indian research, which found that 86% of studies (out of 63) found evidence of a positive link between alcohol and other drug use and historical trauma [36].

A pattern of consistent findings has been identified in qualitative studies with American Indians, identifying that historical trauma contributes to the use of alcohol in their communities [2,3,5,37-40]. Studies conducted by Whitbeck et al [38-40] established that historical loss thinking is prevalent among American Indians and also associated with the use of alcohol. A study by Weichelt et al [41] established that American Indian participants with higher historical loss thinking had a higher likelihood of using alcohol within the past 30 days. Walls and Whitbeck’s [42] study of the multigenerational effects of relocation experiences on Indigenous family groups focused on historical trauma as a contributor to American Indian health disparities, including alcohol use, and to the advantages of treating cultural loss as a source of stress. They examined the multigenerational effects of relocation experiences on American Indian family groups. American Indians living on Indian reservations reported higher levels of drinking problems. The grandparent generations’ relocation experiences were significantly associated with drinking, and the drinking problems were significantly associated with depressive symptoms and other substance use problems. Discrimination was found to be a trigger for historical loss thinking, and perceived discrimination was found to be positively associated with historical losses, which were positively associated with problematic alcohol use in women. Also, many of the traumatic events described by the participants involved both parents and children and were associated with the onset of alcohol misuse [43,44].

### Intergenerational Transmission of Alcohol

The theme of intergenerational influences on alcohol use is consistent with the literature on American Indians. The participants described how historical trauma and problematic alcohol use disrupted their families and their communities and how maladaptive behaviors were maintained and spread through intergenerational historical trauma from the kupuna to the younger generations, undermining healthy family functioning and child development. This is consistent with the literature, which indicates that childhood trauma experiences may impede adults’ abilities to provide responsive caregiving as they lack models of parenting in their own lives [2,45]. Indigenous cultures are interconnected, and generational connections are valued because elders are considered the sources of cultural knowledge. When these connections are broken, the consequences move through subsequent generations [42-44]. The literature shows how the intergenerational cycles of trauma manifest within families, which extends to communities impacted by historical trauma. Walls and Whitbeck’s [42] study on American Indians discussed how the impact of government relocation policies on the grandparent generations’ substance abuse was linked to the subsequent generation female adult caregivers’ depressive symptoms and substance use. As a consequence, the female caregivers were less effective at parenting the next generation, the adolescent children. The substance use problems were negatively associated with supportiveness and warmth. In these circumstances, the grandparent generation functions as a possible risk factor for substance use across generations, and relocation may be a distal source of these problems when framed within the intergenerational model of risk [42]. They demonstrated that the grandparent generation’s participation in government relocation programs negatively impacted not only the grandparent generation’s well-being, leading to depression, alcohol, and other substance use problems, but also subsequent generations’ well-being. Indigenous peoples began using alcohol as a way to self-medicate to cope with emotional pain.

### Limitations

The results of the study should be viewed in light of its limitations. First, the study was conducted in a single rural Hawaii community, with participants being predominantly female, which limits generalizability. Second, while the results reveal similarities in the historical trauma responses to American Indian literature, the findings reflect the perspectives of 10 rural Native Hawaiian participants. While the sample size was adequate to achieve saturation, the findings may not represent the experiences of Native Hawaiians on other Hawaiian Islands or communities in Hawaii. Third, despite data saturation, the number of participants was small. It is uncertain if a larger sample size could have affected the resulting themes. Finally, bias may occur as the study only included those willing to be interviewed. Despite these limitations, findings provided robust insights into a shared history of colonization with American Indians and Native Hawaiians’ perceptions related to historical trauma and alcohol use.

### Implications and Recommendations for Future Research

This study is significant for several reasons and has implications for future research. When it was conducted in 2019, it was the only study that used the historical trauma conceptual model and Native Hawaiian storytelling methodology to explore the impact of colonization and historical trauma among Native Hawaiians. Historical trauma can be transmitted intergenerationally through different mechanisms, such as oral or storytelling transmission, which were explored here.

In addition, it is essential to take into consideration Indigenous groups’ geographical and regional differences in how trauma is experienced and also how individuals within groups experience trauma [23]. For example, the cumulative effects of historical trauma in Native Hawaiians are mitigated by the existence of resiliency and protective factors that are unique to them, requiring additional research. The findings of this qualitative study can be considered as contributing to the current knowledge base on the impact of colonization and historically traumatic experiences as an underlying predictor of alcohol and other substance use among Native Hawaiians. However, multilevel, systemic evaluations are needed to assess Indigenous communities for collective trauma while observing cultural differences between groups. While communities must be evaluated, individual family and community interventions must be developed [23,45-47].

Future research should further expand knowledge related to the health impact of colonization and historically traumatic experiences in diverse context-specific geographic regions. Indigenous groups must drive the research. In addition, healing intervention models such as the historical trauma and unresolved grief intervention adapted to Native Hawaiian experiences should be grounded in Native Hawaiian worldviews to eradicate emotional distress that emerges as a legacy of historical trauma [23]. Currently, the Historical Loss scale and the Historical Loss and Associated Symptoms scale do not include a full measure of depression or post-traumatic stress disorder symptoms and were developed with American Indians [38-40]. New directions in research would be strengthened by expanding and developing new measures tailored to individual Indigenous groups through participatory research, which is currently in progress with Native Hawaiians [22].

### Conclusion

The results of this study provide important insight into Native Hawaiians’ lived experiences of historical trauma and alcohol use. The results characterize the colonizing events that led to historical trauma, the introduction of alcohol, and links to the problem of alcohol use today, the impact, and the mechanisms of transmission. This study reveals commonalities in the historical trauma response, especially the loss of culture, the introduction of alcohol, self-medication with alcohol, and intergenerational transmission of historical trauma and problematic alcohol use from elders to younger generations. The findings can be integrated into the current literature on related occurrences of historical trauma to advance our understanding of the historical trauma response. Necessary next steps include including this knowledge to guide the development of culturally grounded trauma-informed interventions. The long-term goal of historical trauma research is to develop culturally relevant interventions that are informed by the communities to improve the quality of life and empower Indigenous peoples to reclaim traditional knowledge, their identity, and health, and to help heal communities so that they are unburdened by grief over historic losses.

In addition, more context-specific research is needed to validate the historical trauma model, which has implications for healing and prevention. Thoughts and awareness of historical trauma may not be harmful to health if the issues that are associated with them are addressed, and individuals within a group who have experienced the transmission of intergenerational trauma are provided with the resources and tools to manage the stress that is associated with it. Research must fully understand, validate, and operationalize the historical trauma conceptual model’s theoretical constructs and link them to health outcomes. In addition, risk factors, resiliency, protective cultural factors, and survivance must be explored among Indigenous populations to include in the interventions. The knowledge gained from this study can be used to inform policies aimed at addressing historical trauma in Native Hawaiian communities.

## Supplementary material

10.2196/68106 Multimedia Appendix 1 Interview semistructured script.

10.2196/68106 Multimedia Appendix 2 Additional illustrative quotes.
