# Supplementary material for: Exploring a Shared History of Colonization, Historical Trauma, and Links to Alcohol Use With Native Hawaiians: Qualitative Study
Source: Asian Pac Isl Nurs J. 2025 Jul 29;9:e68106. doi: 10.2196/68106 (PMC12306949; doi:10.2196/68106)
Supplement: Multimedia Appendix 1 [file apinj-v9-e68106-s001.docx]

Appendix 1: Interview semi-structured script

| **Interview questions** |
| --- |
| Can you share any stories about alcohol use in Hawai’i before Captain Cook arrived?  Can you share any stories about the kupuna and alcohol use when they were growing up and you were growing up?  Can you share any stories of how alcohol is used by Native Hawaiians in your area currently? |
