# Supplementary material for: Exploring a Shared History of Colonization, Historical Trauma, and Links to Alcohol Use With Native Hawaiians: Qualitative Study
Source: Asian Pac Isl Nurs J. 2025 Jul 29;9:e68106. doi: 10.2196/68106 (PMC12306949; doi:10.2196/68106)
Supplement: Multimedia Appendix 2 [file apinj-v9-e68106-s002.docx]

**Appendix 2:** Additional illustrative quotes

| **Theme** | **Additional illustrative quotes** |
| --- | --- |
| 1. Alcohol did not exist in  Hawaii before European  explorers arrived, | “Alcohol was introduced by Europeans.  “We didn't have alcohol before Captain Cook, and now we do.” |
| 2. Alcohol helped expand  colonialism in Hawaii, | “When our Queen was overthrown, and the lands were taken away, it was taken away with alcohol.”  “Alcohol and the regulation of alcohol by the colonizers before Hawaii became a state was used as a means to help overthrow our monarchy.” |
| 3. Alcohol is used today as a  coping strategy for  feelings of grief and anger  over losses | “NHs drink alcohol to numb feelings of “low self-esteem and being pushed down.”  “People are self-medicating away a lot of pain. It's all because we lost our land. Because somebody else took it, and then they said get out. You're on my land. I don't want you.”  “Unfortunately, people use it as a coping mechanism for their pain about all of the losses we've had...they self-medicate by having a beer, and then all it’s all because we lost our land.” |
| 4. The Kupuna teach the  younger generations to  drink alcohol | “Some of the kupunas drink alcohol and that they are the ones that show everyone else how to drink.”  “Our kupunas teach us how to drink. It's what our kupunas are doing — teaching the younger generation how to drink alcohol and be like that.” |
